# Psychosocial coping with intensive care delirium: a mixed-methods systematic review of patient and family perspectives

**DOI:** 10.1186/s13054-026-06232-1

**Published:** 2026-08-21

**Authors:** Eunkyeong Oh, Gabriella Marx-Rosenberg, Florian Schimböck, Nikolas Groth, Peter Nydahl, Jürgen Gallinat

**Affiliations:** 1 Department of Psychiatry and Psychotherapy, University Medical Center Hamburg-Eppendorf (UKE), Hamburg, Germany; 2 Nursing Research and Development, University Hospital of Schleswig-Holstein (UKSH), Kiel, Germany; 3 Department of General Medicine, Center for Psychosocial Medicine, University Medical Center Hamburg-Eppendorf (UKE), Hamburg, Germany; 4 Institute of General Practice, University Hospital of Schleswig-Holstein/Kiel University, Kiel, Germany; 5 Faculty of Medicine, MSB Medical School Berlin, Berlin, 14197 Germany

**Keywords:** Delirium, Intensive care unit, Coping skills, Family, Systematic review, Patient-centred care, Stress

## Abstract

**Objective:**

This review aims to identify, synthesize, and critically appraise evidence on psychosocial coping strategies employed by intensive care unit (ICU) patients and their family caregivers during an episode of ICU delirium, organized within the transactional model of stress and coping.

**Design:**

Mixed-methods systematic review (MMSR) following the Joanna Briggs Institute (JBI).

**Information sources:**

A systematic search was conducted in MEDLINE (PubMed), CINAHL, PsycINFO, and PSYNDEX. The literature search concluded on the 6th of November 2025.

**Methods:**

Following JBI methodology for mixed-methods systematic reviews, qualitative findings were analyzed using the JBI meta-aggregation approach. Methodological quality was assessed using the Mixed-Methods Appraisal Tool (MMAT). The transactional model of stress and coping served as the theoretical framework. Reporting followed the Preferred Reporting Items for Systematic Reviews and Meta-Analyses (PRISMA) 2020 statement.

**Results:**

Ten qualitative studies were included, originating from seven countries, published between 1999 and 2025. No quantitative studies meeting the eligibility criteria were identified, representing a significant gap in literature. Patients employed problem-focused coping strategies (active sense-making, reality orientation, proactive avoidance of delirium triggers), emotion-focused strategies (social connection, distancing, self-control), and meaning-based strategies (positive reappraisal, identity restoration). Families directed coping efforts both at supporting the patient through emotional presence, active reorientation, cognitive stimulation, and collaboration with the healthcare team, and at managing their own distress through information-seeking, avoidance, and positive reappraisal.

**Conclusions:**

Both ICU patients and their families actively employ a broad range of coping strategies in response to delirium, consistent with the transactional model of stress and coping. Family caregivers emerge as a primary coping resource for patients while simultaneously managing their own psychological burden. These findings suggest that coping strategies may directly influence the trajectory of delirium-related psychological distress, and provide an evidence base for developing patient- and family-centered, coping-informed psychosocial interventions in ICU delirium care.

**Supplementary Information:**

The online version contains supplementary material available at 10.1186/s13054-026-06232-1.

## Introduction

Delirium is an organic brain syndrome with acute fluctuation of the state of consciousness and attention [1]. This complex neuropsychiatric syndrome is a prevalent condition among medical inpatients. In adult intensive care units (ICU), the incidence ranges from 31% up to 80% [2]. During the COVID-19 pandemic, delirium prevalence among ICU patients reached 54.9% in a large multicentre cohort across 14 countries [3]. It has significant negative impacts on cognition, mortality, functional outcomes, quality of life, and the likelihood of long-term institutionalization after hospitalization [4]. Since older adults are disproportionately vulnerable to delirium, population ageing is expected to increase the clinical burden of this condition [5].

From a psychological perspective, ICU admission represents a profound stressor for both patients and their families (i.e., partners, parents, children, close family caregivers, and other significant persons emotionally involved in the patient’s care) [6]. Schmitt et al. (2019) identified three main burden dimensions shared across patients, family caregivers, and nursing staff, that are frequently accompanied by delirium: symptom burden, emotional burden and situational burden, reflecting that no single group experiences delirium in isolation [7].

Patients experiencing delirium frequently report intense emotional symptoms, including fear, anger, shame and helplessness, alongside cognitive disturbances such as hallucinations and disorientation [8, 9]. Beyond the acute episode, ICU delirium is associated with long-term psychological sequelae including depression, anxiety, post-traumatic stress disorder (PTSD), and cognitive impairment, which substantially compromise survivors’ health-related quality of life [10–15].

Family caregivers of ICU patients also face multiple psychosocial stressors. Difficulty understanding medical care, communication barriers with healthcare providers, and overwhelming emotional responses are often marked by guilt, grief, and emotional numbness [16]. Furthermore, witnessing a loved one in a delirious state is associated with anxiety, depression, and PTSD [17].

Notably, no single group experiences delirium in isolation; it constitutes a shared stressor affecting multiple stakeholders [7]. ICU patients and families identified emotional distress as the single most important outcome of delirium to be addressed in research, ranking it first among 100 possible outcomes [18].

Lazarus and Folkman’s transactional model of stress and coping conceptualizes coping as the active cognitive and behavioral efforts employed to manage demands appraised as stressful or exceeding one’s resources, evaluated through continuous appraisal and reappraisal processes [19]. Within this framework, coping responses can be organized into three categories: problem-focused coping, directed at managing the source of stress; emotion-focused coping, aimed at regulating the emotional response; and meaning-based coping, encompassing positive reappraisal and the reconstruction of meaning under conditions of enduring stress [20–22]. Beyond their psychological function, coping strategies are clinically relevant, as the way individuals manage stress has been shown to carry measurable physiological consequences for health and recovery outcomes [23, 24]. Applied to ICU delirium, this framework offers a structured lens through which the active responses of patients and families to one of the most distressing experiences in critical care can be systematically examined. The coping strategies that patients and families employ in response to ICU delirium may directly shape the severity and trajectory of these psychological outcomes: whether fear, helplessness, or distress lead to lasting psychological sequelae or whether they are processed and integrated into recovery. Therefore, this framework not only contextualizes the psychological burden of ICU delirium but serves as the primary analytical structure guiding the synthesis and interpretation of coping strategies identified in this review.

Despite the substantial psychological burden imposed by ICU delirium on patients and their families, the coping strategies employed by both groups have not been systematically investigated. Existing qualitative research has primarily documented the delirium-related distressing experiences of patients and families, encompassing emotional, cognitive, physical, and relational suffering [25]. While some studies have identified individual coping responses, such as avoidance, confrontation, and sense-making, in patients recovering from ICU-related experiences [10, 26], a systematic synthesis of the active coping processes employed by both patients and families specifically in response to ICU delirium remains lacking. Understanding coping is particularly relevant here, as it captures the active processes through which patients and families navigate the experience itself, and thus constitutes a necessary precondition for developing targeted psychosocial interventions. This review therefore addresses this gap by providing the first systematic synthesis of coping strategies from the bilateral perspective of patients and their families.

This mixed-methods systematic review aims to identify, synthesize, and critically appraise the available evidence on coping strategies employed by ICU patients and their family caregivers during ICU delirium. The findings are intended to contribute to the development of more targeted psychosocial support strategies for both groups in delirium care in clinical practice.

## Objectives

The goal of this review was to identify, synthesize, and critically appraise evidence on the psychosocial coping strategies employed by ICU patients and their family caregivers in response to delirium, organized within the transactional model of stress and coping. It aimed to identify problem-focused, emotion-focused, and meaning-based coping strategies from both perspectives and to explore the relational processes between the two.

## Methods

Adhering to the Joanna Briggs Institute (JBI) methodology for mixed-methods systematic reviews, this study complies with the *Preferred Reporting Items for Systematic Reviews and Meta-Analyses (PRISMA)* 2020 reporting standards. The review process was initiated in 2025 and prospectively registered in the PROSPERO database (registration number: CRD420251186487).

### Eligibility criteria

#### Inclusion criteria

Studies exploring coping related to the ICU delirium experience were eligible for inclusion if they met the following criteria:

- **P**opulation: Adult patients ($\:\ge\:$18 years) who experienced or were reported to have experienced delirium during an ICU stay, and/or family caregivers of these patients (i.e., partners, parents, children, close family caregivers, and other significant persons emotionally involved in the patient’s care, including chosen family structures).
- **I**ntervention: any psychosocial intervention targeting coping with ICU delirium operationally defined as any structured psychological, social, or supportive intervention explicitly aimed at facilitating adaptive coping responses in ICU patients or their families during a delirium episode (for the quantitative component).
- **P**henomena of Interest: psychosocial coping strategies in response to ICU delirium, including problem-focused, emotion-focused, meaning-based, spiritual, and social coping, as well as resilience, adaptation, and psychological recovery (for the qualitative component).
- **O**utcomes: Empirical measures of coping, resilience, or psychological recovery (quantitative component).
- **C**ontext: Intensive care units (ICU, CCU, ITU, Critical Care); studies conducted during ICU stay or using retrospective accounts of the ICU delirium experience were considered (qualitative component).
- Time frame: Publications up to 6th of November 2025.

Qualitative, quantitative, and mixed-methods studies conducted during the patients’ ICU stay, after ICU discharge, or in post-intensive care recovery settings were considered, provided a clear link to the delirium episode existed. There was no language restriction applied to the search process.

#### Exclusion criteria

Studies conducted outside of an adult ICU context, including pediatric, neonatal, or palliative care settings, were excluded. Research centered on caregivers or healthcare professionals without a direct link to the patient’s delirium experience was deemed ineligible. Studies addressing only delirium prevention or purely pharmacological and physiological management were excluded, unless a clear psychosocial coping focus existed. Articles focusing solely on neurobiological mechanisms or cognitive function in isolation were not considered eligible for inclusion, as they did not address the psychosocial coping processes that formed the primary focus of this review. Non-empirical publications, including reviews, meta-analyses, protocols, commentaries, and purely theoretical discussions, were excluded from this synthesis. Conference abstracts without full texts, case series, and gray literature such as theses and conference papers were also excluded.

### Search strategy

A systematic search was performed across four electronic databases: MEDLINE (PubMed), CINAHL, PsycINFO, and PSYNDEX, using key search terms including ‘delirium’, ‘intensive care’, ‘coping’, and ‘family’. Detailed search strings and strategies are provided in the Supplementary Material (Additional File 1). No time restrictions were applied to the search to ensure a comprehensive overview of the available evidence. The literature search concluded on the 6th of November 2025. This review relied exclusively on existing literature; no primary data were collected.

**Fig. 1:**
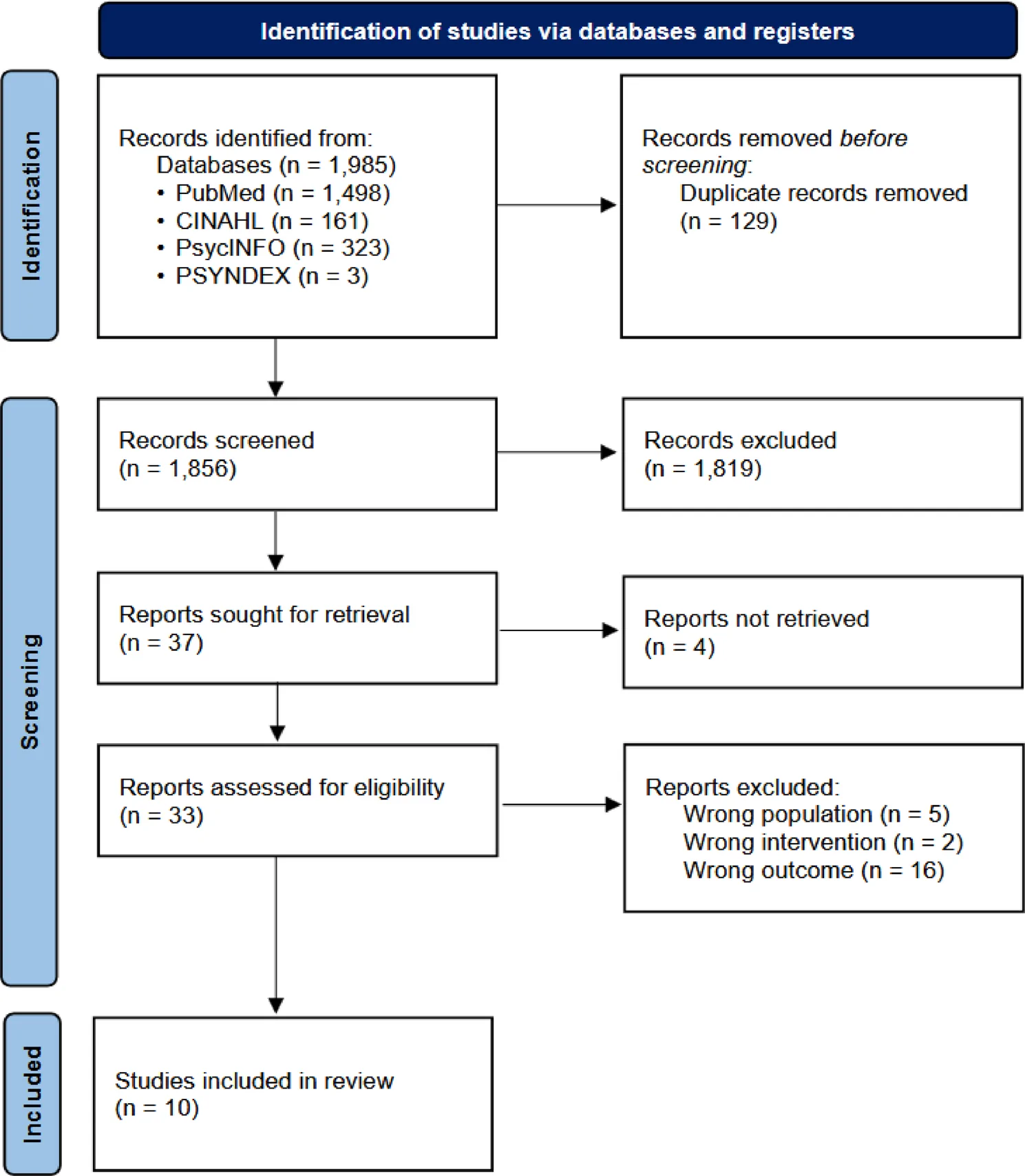
PRISMA flow diagram of the study selection process. The flowchart illustrates the stages of literature identification, screening of titles and abstracts, full-text eligibility assessment, and the final inclusion of studies for the systematic review

### Study selection process

Initial search results were exported to the Rayyan platform for screening [27]. Following de-duplication, two investigators (E.O., F.S.) independently evaluated all titles and abstracts against the eligibility criteria between November and December 2025. Full-text versions were retrieved for any record deemed potentially relevant by at least one reviewer. Final inclusion was determined after a secondary screening of these full texts. During full-text screening, disagreements occurred for eight of 33 assessed records (24%), all of which were resolved through consensus discussion between the two reviewers. The selection workflow is visualized in the PRISMA flow diagram (Fig. 1).

### Quality assessment of publications

The methodological quality of the included studies was assessed independently by two investigators (E.O., N.G.) between December 2025 and March 2026, using the *Mixed-Methods Appraisal Tool (MMAT)* [28]. Each study was appraised on the basis of five design-specific criteria. Although the MMAT does not recommend calculating an overall score or ranking, for narrative purposes in this review, studies with one or no criteria rated as “No” or “Can’t tell” are referred to as higher-quality, and those with two to three such ratings as moderate-quality. To avoid overstating study robustness, these classifications should be interpreted cautiously. Conflicts in the quality assessment were resolved through mutual dialogue. No external clarification from study authors or a third reviewer was required.

### Data extraction

Data extraction was performed independently by two reviewers (E.O., N.G.) between December 2025 and March 2026, using a data extraction form developed by the review team. Extracted information included author, year, country, study design, setting, participant characteristics including sample size, reported outcomes, and coping strategies identified from both patient and family perspectives.

### Data synthesis and integration: convergent-integrated approach

This mixed-methods systematic review followed the JBI *convergent integrated approach* to data synthesis [29]. The search strategy was employed to capture both quantitative and qualitative evidence on coping strategies in ICU delirium. However, following the systematic screening process, no quantitative studies meeting the eligibility criteria were identified. The synthesis therefore was implemented exclusively on qualitative evidence. The MMSR framework is nonetheless retained, as the review was designed and prospectively registered as a mixed-methods systematic review in prior to the search. The absence of quantitative evidence is therefore a finding of the review process rather than a post-hoc modification of the design.

Qualitative findings were analyzed using the JBI meta-aggregation approach, comprising three stages: (1) extraction of findings and assessment of their credibility, (2) categorization of findings into descriptive categories, and (3) synthesis of categories into synthesized findings [29]. The categorization was conducted following a deductive-inductive approach. Findings were primarily organized according to the transactional model of stress and coping [19, 21], while categories emerging beyond this framework were incorporated inductively. In the final step, all synthesized findings were integrated into a comprehensive synthesis, linking the identified coping strategies with participant experiences and contextual factors.

## Findings

The systematic search across the electronic databases yielded 1,985 citations. Following the removal of 129 duplicates, 1,856 records were screened by title and abstract. Thirty-seven full-text articles were sought for retrieval, of which four could not be obtained; 33 were assessed for eligibility. After full-text evaluation, 23 papers were excluded: five due to wrong population, two due to wrong intervention, and 16 due to wrong outcome. Ten studies met all eligibility criteria and were included in the final synthesis.

An overview of the study selection process is illustrated in the PRISMA flow diagram (Fig. 1). The extracted data from each included study are provided in Table 1 as an additional file (Table 1), forming the basis for the synthesis of coping strategies in ICU delirium from the bilateral perspective of patients and their families.

**Table 1:** Characteristics of the included studies

Author, year (country)	Qualitative design	ICU-Type/population	Data collection & analytic method	Coping: primary/secondary & context	Coping strategies (patients / family)
Granberg et al., 1999 (Sweden)	Hermeneutic phenomenology	General ICU / mechanically ventilated patients	Semi-structured interviews, *n*=19, hermeneutic text analysis (Malterud)	Secondary / emergent finding Context: Post-ICU recovery	Patients: - Cognitive coping: trying to make sense of unreal experiences; denying symptoms of delirium; mental relocation to pleasant places (hope, conviction of survival) - Behavioral coping: seeking support from relatives; internal dialogue - Emotion-focused: managing fear through trust and confidence in caregivers Family: - Not explicitly analyzed; mentioned indirectly as stabilizing and reassuring factor
Whitehorne et al., 2015 (Canada)	Hermeneutic phenomenology (van Manen)	Medical/Surgical ICU / CAM-ICU positive patients	Semi-structured interviews, *n*=10, post-ICU transfer; van Manen thematic analysis	Secondary / emergent finding Context: Post-ICU recovery	Patients: - Cognitive coping: searching for cues to understand surroundings; reality-testing - Behavioral coping: connecting with staff; avoiding triggers of further delirium (e.g. refusing sedation) - Emotion-focused: familiar objects (e.g. a cross); feeling cared for through touch and kind words Family: - Offering familiar objects to provide sense of safety
Darbyshire et al., 2016 (UK)	Qualitative secondary analysis / thematic analysis	Mixed ICU / general ICU patients	Secondary analysis of archived interviews, *n*=40 patients, *n*=37 family members; thematic analysis (constant comparison)	Secondary / emergent finding Context: Post-ICU recovery (retrospective)	Patients: - Cognitive coping: creating narratives; deliberate attempts to make sense of delirium-related delusions and disorientation - Behavioral coping: actively trying to avoid sleep to escape dreams - Emotion-focused: realizing being in ICU as reassuring Family: - Not explicitly analyzed
Smithburger et al., 2017 (USA)	Grounded theory	Medical ICU, academic centre / family members of delirium patients	Telephone interviews, *n*=10; grounded theory coding (NVivo)	Secondary / emergent finding Context: Family caregiving during ICU stay	Patients: - Not explored Family (directed at patient): - Presence and physical touch (holding hands); emotional support; hygiene activities; comfort measures; reorientation; cognitive stimulation - Bringing familiar objects (whiteboard, books, calendar, pillow) Family (managing own distress): - Open communication with healthcare providers; information-seeking about delirium prevention; collaborative coping with healthcare team
Bohart et al., 2019 (Denmark)	Phenomenology (Malterud STC)	General medical/surgical level II ICU / family members of CAM-ICU positive patients	Semi-structured interviews, *n*=11, during/shortly after ICU admission; systematic text condensation (Malterud)	Secondary / emergent finding Context: Family caregiving during ICU stay	Patients: - Not explored Family (directed at patient): - Adapting communication; accepting rather than correcting; calm presence - Collaborative coping with healthcare team Family (managing own distress): - Normalization of delirium (prioritizing survival over cognition) - Information-seeking; hopeful outlook despite uncertainty - Selective engagement: letting minor issues go
Huang et al., 2022 (Taiwan)	Descriptive qualitative study	Adult ICU, medical centre northern Taiwan / family caregivers of delirium patients	Face-to-face semi-structured interviews, *n*=20 family caregivers, ~1 h each; qualitative content analysis	Secondary / emergent finding Context: Family caregiving; timing relative to ICU discharge not explicitly stated	Patients: - Not explored Family (managing own distress): - Religious rituals (praying, divination); traditional Chinese medicine (CAM) - Information-seeking from other families about folk remedies - Providing emotional support during restricted visitation - Passive trust in staff due to lack of delirium information - Sense-making of patients’ treatment (e.g. physical restraint)
Ma et al., 2024 (China)	Descriptive qualitative study	Step-down unit, tertiary hospital / post-ICU delirium patients and families	Face-to-face interviews, *n*=17 (6 patients, 11 family members; 6 dyads + 5 individual); inductive thematic analysis (Braun & Clarke), NVivo 11 Plus	Secondary / emergent finding Context: Post-ICU transfer; interviews during ongoing hospitalization	Patients: - Not explored Family (directed at patient): - Reality orientation; emotional support (positive messages, encouragement) - Linking patients to outside world (family routines, rebuilding social roles) - Improving sense of security; alleviating psychological burden - Encouraging trust in medical staff and active cooperation
Menza et al., 2024 (USA)	Grounded theory (Charmaz)	Urban academic Level-1 trauma centre; trauma/neuro ICU / patients, family members, friends	Semi-structured interviews, *n*=14 interviews / 21 informants; grounded theory (Charmaz), open coding, constant comparison (Atlas.ti 9.1)	Secondary / single code only; not a named theme Context: Predominantly during ICU/hospital stay; 1 interview 7 months post-admission	Patients: - Music as sense-making and reality grounding; feeling of being brought back to themselves - Regaining control and autonomy through music choice - Distraction from ICU environment sounds - Connecting with identity and belief systems; restoring sense of purpose - Addressing psychological wellbeing: managing intrusive thoughts, processing emotions Family: - Using music to restore consciousness and trigger memories - Providing connection to life outside hospital; lessening loneliness - Resolving problems of silence; preventing disorientation
Dong et al., 2025 (China)	Qualitative descriptive study (naturalistic inquiry)	3 mixed ICUs, tertiary hospital / post-operative delirium patients and families	Dyadic semi-structured interviews, 18 patient-family pairs + 2 additional family members, within 1st week post-ICU discharge; thematic analysis (Braun & Clarke), NVivo 12	Secondary / emergent finding Context: Post-ICU discharge, within first week after transfer	Patients: - Family presence as source of comfort and motivation to survive - Emotional bond to family as spanning illness and circumstances - Communicating with family about the post-operative delirium experience Family (directed at patient): - Extended presence (face-to-face and video calls) - Acting as communication mediators between staff and patient - Summarizing coping methods based on personal knowledge of patient Family (managing own distress): - Avoiding transfer of emotional burden to patient - Actively seeking help from multiple perspectives - Maintained balance between downplaying delirium memories and unravelling confusion
Zhang et al., 2025 (China)	Interpretative Phenomenological Analysis (IPA)	15-bed ICU, tertiary hospital / elderly post-operative patients (≥65 years) and families	Semi-structured individual interviews, 8 patient-family dyads, one month post-ICU discharge, 30–45 min; IPA (NVivo 15)	Primary / explicitly named theme (“Finding Ways to Cope”); not theoretically pre-specified Context: Post-ICU discharge, one month after transfer	Patients: - Not explored Family (managing own distress): - Self-protection: avoiding discussions about delirium to prevent distress/embarrassment - Optimism about home recovery - Reaching out for professional support (talking to psychologist)

The extracted data included author(s), year, country, study design, population, outcomes, and coping strategies from patient and family perspectives. Data extraction was conducted independently by two reviewers (E.O., N.G.) following the JBI guidelines for mixed-methods systematic reviews

ICU - intensive care unit; POD - post-operative delirium; CAM - complementary and alternative medicine

### Characteristics of included studies

Ten qualitative studies were included in this review, published between 1999 and 2025 and originating from seven countries. The majority of studies were conducted in China (*n* = 3) [30–32], followed by single studies from Sweden [33], Canada [34], the United Kingdom [35], the United States of America [36, 37], Denmark [38], and Taiwan [39].

Regarding study population, five studies included both patients and family caregivers [30–32, 35, 36], three focused exclusively on family caregivers [37–39], and two investigated patients only [33, 34]. Sample sizes ranged from six to 77 participants. All studies were conducted in adult ICU settings, with the majority focusing on post-operative or medical ICU contexts. Overview of the study characteristics is provided in Table 1.

### Methodological quality assessment

The overall methodological quality of the included studies was high. Among the ten included qualitative studies, 80% (*n* = 8/10) were classified as higher-quality, fulfilling all five MMAT criteria [30–34, 36, 38, 39], and 20% (*n* = 2/10) had “Can’t tell” rating on one criterion [35, 37].

For all eligible qualitative studies, the type of study design was assessed as adequate. Findings were clearly grounded in the data, providing a coherent analysis and interpretation of the results substantiated by the data. Darbyshire et al. (2016) had a “Can’t tell” rating on data collection methods, as the procedures were not described with sufficient clarity. Smithburger et al. (2017) received a “Can’t tell” rating on the derivation of findings, as the link between raw data and reported results was not fully transparent [35, 37].

The quality assessment results are presented in detail in Table 2 (additional file Table 2).

**Table 2:** Critical appraisal results of eligible studies using the mixed-methods appraisal tool (MMAT)

Author, year	Critical appraisal of eligible qualitative studies					
	Q1. Is the qualitative approach appropriate to answer the research question?	Q2. Are the qualitative data collection methods adequate to address the research question?	Q3. Are the findings adequately derived from the data?	Q4. Is the interpretation of results sufficiently substantiated by data?	Q5. Is there coherence between qualitative data sources, collection, analysis and interpretation?	
Granberg et al., 1999	Y	Y	Y	Y		Y
Whitehorne et al., 2015	Y	Y	Y	Y		Y
Darbyshire et al., 2016	Y	CT	Y	Y		Y
Smithburger et al., 2017	Y	Y	CT	Y		Y
Bohart et al., 2019	Y	Y	Y	Y		Y
Huang et al., 2022	Y	Y	Y	Y		Y
Ma et al., 2024	Y	Y	Y	Y		Y
Menza et al., 2024	Y	Y	Y	Y		Y
Dong et al., 2025	Y	Y	Y	Y		Y
Zhang et al., 2025	Y	Y	Y	Y		Y

Methodological quality was assessed independently by two reviewers (E.O., N.G.) via the Mixed Methods Appraisal Tool (MMAT, version 2018). Each study was evaluated according to five design-specific criteria. Following MMAT guidance, studies were not scored numerically but categorized descriptively as high, moderate, or low quality for narrative purposes

MMAT - Mixed Methods Appraisal Tool; Y - yes (criteria met); N - no (criteria not met); CT - can’t tell (unclear or insufficient information)

### Review findings

#### Coping strategies: patient perspective

A broad range of coping strategies was identified in response to ICU delirium from inpatient perspectives, categorized in problem-focused, emotion-focused, and meaning-based coping as conceptualized by Lazarus and Folkman [19].

The most common approach was active sense-making and reality orientation. Through searching for cues in the environment to understand what was happening and where they were, patients tried to support sense-making of their surroundings and ground their perceptions in reality [34, 36]. Creating narratives about the situation were deliberate attempts to gain understanding of the reality [35]. Similarly, patients attempted to find explanations for unreal experiences as a means of cognitive mastery [33]. Identified in four of the included studies, this problem-focused coping pattern is the most prominent acute strategy of patients.

Further problem-focused coping skills included the regaining of control, autonomy and the initiating of proactive avoidance behaviors. Despite the predominantly constrained role patients occupy during ICU hospitalization, some actively sought to regain control through choosing and playing the music by themselves or moderating sudden sounds of monitors and doors that were disturbing [36]. Patients also actively rejected moments that might trigger a further delirium episode, refusing sleeping pills or sedation [34].

Following the efforts of sense-making, reaching out for social connection was consistently evidenced across multiple included studies. Patients were seeking support from family caregivers, in which family presence provided comfort and the strength to survive [30, 33]. The feeling of being cared for through kind words or physical touch provided emotional bond to family [34]. Furthermore, communicating with family caregivers about delirium helped patients make sense of their symptoms and the circumstances [30]. In addition, connecting with staff improved feelings of safety and being in touch with reality [34]. Amongst the emotion-focused coping resource, social connection emerged as the most consistently reported approach, reflecting the central role of interpersonal relationships in the secondary appraisal process [19].

Distancing and avoidance were also frequently described emotion-focused coping strategies. Some patients denied symptoms of delirium because they believed they were going crazy [33]. Others tried to distract themselves from mechanistic sounds in ICU or to take their mind off problems such as anxiety, breathlessness, and frustration [36]. One patient described actively trying to avoid sleep to escape dreams [35].

Another pattern of coping as an emotion-focused strategy was self-control. In order to manage the feelings of fear, patients tried to process their own emotions and to control their intrusive thoughts [36], sometimes by keeping internal dialogues [33]. Having trust and confidence in the caregivers or possessing a familiar object also provided them a feeling of safety as a way of accessing resilience during the ICU stay with delirium [33, 34].

Finally, as a form of meaning-based coping, positive reappraisal and identity restoration were reported frequently. Besides the effort of sense-making, described above as problem-focused coping, patients tried mental relocation to pleasant places and situations, encouraging their hope and conviction of survival in fearful situations [33]. Familiar music was also reported as delivering a feeling of being brought back to themselves, when aligning with individual or family identity. It supported restoring consciousness and helped to experience hope and pleasure [36]. One study reported spiritual beliefs and practices connecting patients with their belief systems, restoring a sense of purpose [36]. These meaning-based strategies are consistent with Folkman's (1997) conceptualization of positive reappraisal and spiritual beliefs as adaptive coping processes that sustain positive psychological states even under conditions of severe and enduring stress [21].

Overall, patient coping with ICU delirium encompassed problem-focused, emotion-focused, and meaning-based strategies, with active sense-making and social connection representing the most consistently evidenced approaches across the included studies.

#### Coping strategies: family perspective

Coping strategies of family caregivers are distinguished into two different directions. One is in supporting and assisting the patient, the other is managing one’s own distress, as specified below separately.

##### A. Coping strategies directed at the patient

The most prominent coping strategy was emotional presence and relational support. Families stayed present at bedside, initiating physical touch with the patients [37]. Their calming speech provided personalized encouragement and motivation, conveying messages that the family is well and not to worry about economic issues [31, 38]. Communication contents invoking memories of life outside hospital built a source of connection to the normal life for the patients [36]. By addressing psychological needs, they supported alleviating patients’ psychological burdens [31]. Families sometimes lessened patients’ loneliness through music-related memories or offered familiar objects to provide the feeling of safety even when they left the visiting time [34, 36]. During times of restricted visitation, positive messages functioned as emotional support, adding to patients’ feeling of security and relaxation [31, 39]. This type of emotion-focused coping was identified in more than half of the included studies, representing the strongest evidence among family coping strategies directed at patients.

In line with the emotion-focused coping, families also reported deploying self-control. Family caregivers were cautious and avoided the transfer of their emotional loads to patients. They maintained balance between downplaying patients’ memories of delirium and unravelling the confusion of patients’ changes [30]. As an act of self-regulation, they tried to be in selective engagement by letting minor issues go, accepting the reality rather than correcting them [38].

The second most frequently reported coping strategy directed at patients was active reorientation and cognitive stimulation. Families tried to provide reality orientation through various ways [31]. They brought items from home - such as books, calendars, and familiar objects - creating home-like familiarity and reducing patients’ anxiety. Families also repeatedly explained time, place and situations to aid communication and patients’ reorientation [37]. Some family caregivers suggested items and checklists as potential methods to facilitate interaction [37]. Another way of supporting patients’ navigation through delirium was through cognitive engagement [36]. For example, cognitive function was maintained through sensory stimulus such as music, triggering memories and initiating awakening of patients [36]. Music was also helpful in providing feeling of safety [36]. One study reported extended family presence through video-call visitations to improve patients’ mental status and support the delirium treatment adherence [30]. These problem-focused coping strategies were not formally mandated as institutional guidelines, but initiated voluntarily by family caregivers instead, contributing to cognitive reorientation as well as to social connection [37].

Collaboration with the healthcare team was reported as another problem-focused coping strategies. Family caregivers proactively initiated open communication and collaboration with healthcare providers in taking care of their loved one [37]. Acting as communication mediators between staff and patients, they put effort into adapting the communication, encouraging patients to trust medical staff and cooperate actively [30, 31]. Similarly, families actively sought help from different perspectives when concerned about patients’ states [30]. With regard to information-seeking for patient care, families also sought education on delirium prevention and management [37]. Besides the collaboration with the medical team, looking after information from other families about folk remedies or the use of alternative treatments like Chinese complementary and alternative medicine (CAM) were also reported [39].

Lastly, as meaning-based coping strategies, reconstruction of identity was described. By assisting recall and reconstruction, families intended to rebuild former social roles, linking patients to the outside world through reporting family routines and events [31]. Kinship and unique understanding of the person’s personality and preferences comforted patients [30]. Invoking other memories of life outside the hospital contributed to the identity reconstruction as well [36]. As mentioned in providing familiarity, playing music was also helpful in getting patients back to the music-related memories, restoring their consciousness [36]. This collaborative involvement of families in reconstructing patients’ identity and restoring their connection to life outside the ICU is consistent with [21] conceptualization of positive reappraisal as strategies, sustaining positive psychological states under conditions of severe stress [21].

In summary, emotional presence and relational support represented the most consistently deployed strategy, active reorientation and identity reconstruction underlining the role of families as active coping agents in delirium care. Family coping strategies directed at the patient reflect the transactional nature of the stress and coping process [19].

##### B. Coping strategies directed at managing their own distress

Family caregivers not only directed the coping efforts towards the patients, but also employed strategies to manage their own psychological distress in response to delirium experience.

Information-seeking and professional support represented the most frequently reported problem-focused coping strategy. Families sought more information about delirium to better understand and manage the situation, and open communication with healthcare professionals was beneficial and relieving [37, 38]. One study reported that families reached out for professional psychological support by talking to a psychologist [32].

Avoidance and self-protection were equally frequently reported emotion-focused coping strategies. Some family caregivers passively trusted the medical staff due to a lack of information regarding delirium and its treatment [39]. Others avoided discussions about delirium to prevent their own distress and embarrassment [32]. One study described families maintaining a careful balance between downplaying patients’ delirium memories and unravelling the confusion of patients’ changes as a protective strategy for both parties [30].

In addition, self-control was reported as another emotion-focused strategy. Families managed their own stress through hope and reassurance, practicing selective engagement by letting minor issues go [38].

Normalization and positive reappraisal constituted meaning-based coping strategies in two studies. Families described a hopeful outlook despite uncertainty, prioritizing survival over cognition as a way of normalizing the delirium experience [38]. Similarly, optimism about home recovery was reported as a coping response to the clinical situation [32].

Finally, spiritually and culturally embedded coping was identified in one study. Families engaged in religious rituals such as praying and divination and utilized traditional Chinese medicine to manage their distress [39]. Although supported by limited evidence, these findings point to the cultural specificity of coping processes in ICU delirium.

Overall, family coping with ICU delirium spanned emotion-focused, problem-focused, and meaning-based strategies in both directions: supporting the patient and managing own distress. Emotional presence and relational support represented the most consistently described approach directed at the patient, whereas information-seeking and avoidance were the predominant strategies families employed to cope with their own psychological burden.

## Discussion

This mixed-methods systematic review synthesized evidence of coping strategies employed by ICU patients and family caregivers in response to delirium experience from ten qualitative studies. Rather than being passively exposed to the experience, both patients and families actively employed a broad range of coping strategies during ICU delirium, encompassing problem-focused, emotion-focused, and meaning-based patterns. For patients, active sense-making and social connection were the most consistently evidenced approaches. Family caregivers directed coping efforts both at supporting the patient, through emotional presence, active reorientation, and collaboration with the healthcare team, and at managing their own distress through information-seeking, avoidance, and positive reappraisal.

These findings align with previous evidence categorizing delirium burden in three main patterns from a multilateral scope. Patients, families and healthcare professionals mainly reported symptom burden, emotional burden and situational burden [7]. Accordingly, this review reveals the prominent deployment of problem-focused and emotion-focused coping strategies, synthesizing the active engagement of patients and family caregivers in response to ICU delirium. In addition, parallel to a study with informal caregivers’ coping strategies of ICU coma patients, describing maladaptive coping patterns such as avoidance or distraction as well as adaptive ones such as acceptance or meaning making, this review data mirrors these behavioral patterns of families of ICU delirium [16]. Whereas existing evidence documented the variety of stressors in regard to delirium in a critical care setting [25], this review goes beyond documenting stressors by synthesizing the active coping strategies employed in response to ICU delirium from a bilateral perspective. Families acted as the main coping resource and patients, despite their constrained role during ICU admission, demonstrated problem- and emotion-focused strategies, forming a complementary bilateral coping response. Therefore, this review addresses a significant gap, providing the first systematic synthesis of the active coping processes through which patients and families navigate ICU delirium.

The data also suggest boundaries to adaptive coping. When families lacked sufficient information, passive trust replaced active engagement [39], and some resorted to avoidance and self-protection when the psychological burden became overwhelming [32]. Families who took on excessive caregiving roles risked emotional exhaustion [38], and consciously avoided transferring their own distress to patients to protect the supporting relationship [30].

### Application and boundaries of the transactional stress model

The synthesized findings indicate that coping with ICU delirium is not a linear but a dynamic process, consistent with the transactional model of stress and coping [19]. ICU delirium constitutes a stressful person-environment transaction [19], triggering continuous appraisal and reappraisal processes in both patients and families [30]. This is consistent with Folkman’s (1997) conceptualization of meaning-based coping as emerging under prolonged stress when primary coping efforts prove insufficient [21].

Furthermore, consistent with Folkman and Lazarus (1988), coping and emotional experience operated in a bidirectional relationship [20]. Sense-making contributed to reducing patients’ disorientation and anxiety [33, 34], while seeking social connection altered emotional states positively [30].

Despite the cognitive constraints imposed by delirium, patients evidenced meaning-based coping strategies including positive reappraisal, identity restoration, and spiritual beliefs [33, 36]. Families similarly engaged in normalization and identity reconstruction as meaning-based processes [30, 38]. That both groups sustained positive psychological states under severe stress is consistent with Folkman’s (1997) conceptualization of meaning-based coping as an adaptive response to enduring stress [21].

However, applying the transactional model of stress to ICU delirium coping reveals an important theoretical consideration. Given the acute cognitive impairment characteristic of delirium, patients may not have been fully capable of deliberate appraisal processes as conceptualized in the model. This may partly explain the lower evidence density of patient coping strategies compared to those of family caregivers, who were cognitively unimpaired and thus better positioned to engage in conscious, goal-directed coping. That patients nonetheless demonstrated active coping, including meaning-based strategies such as positive reappraisal and identity restoration, suggests that coping capacity is not fully suspended even in states of impaired consciousness, and that some strategies may emerge reactively or below the threshold of deliberate cognitive processing. This distinction warrants further investigation, particularly in post-ICU contexts where cognitive capacity is restored.

Additionally, not all coping behaviors identified in this review are explainable through this model. Other than spiritual beliefs, explicitly included in Folkman’s (1997) extended model [21], culturally embedded strategies such as divination, folk and traditional medicine, extend beyond its scope [39]. These findings underline the need to consider cultural background when assessing and supporting coping in clinical practice.

**Fig. 2:**
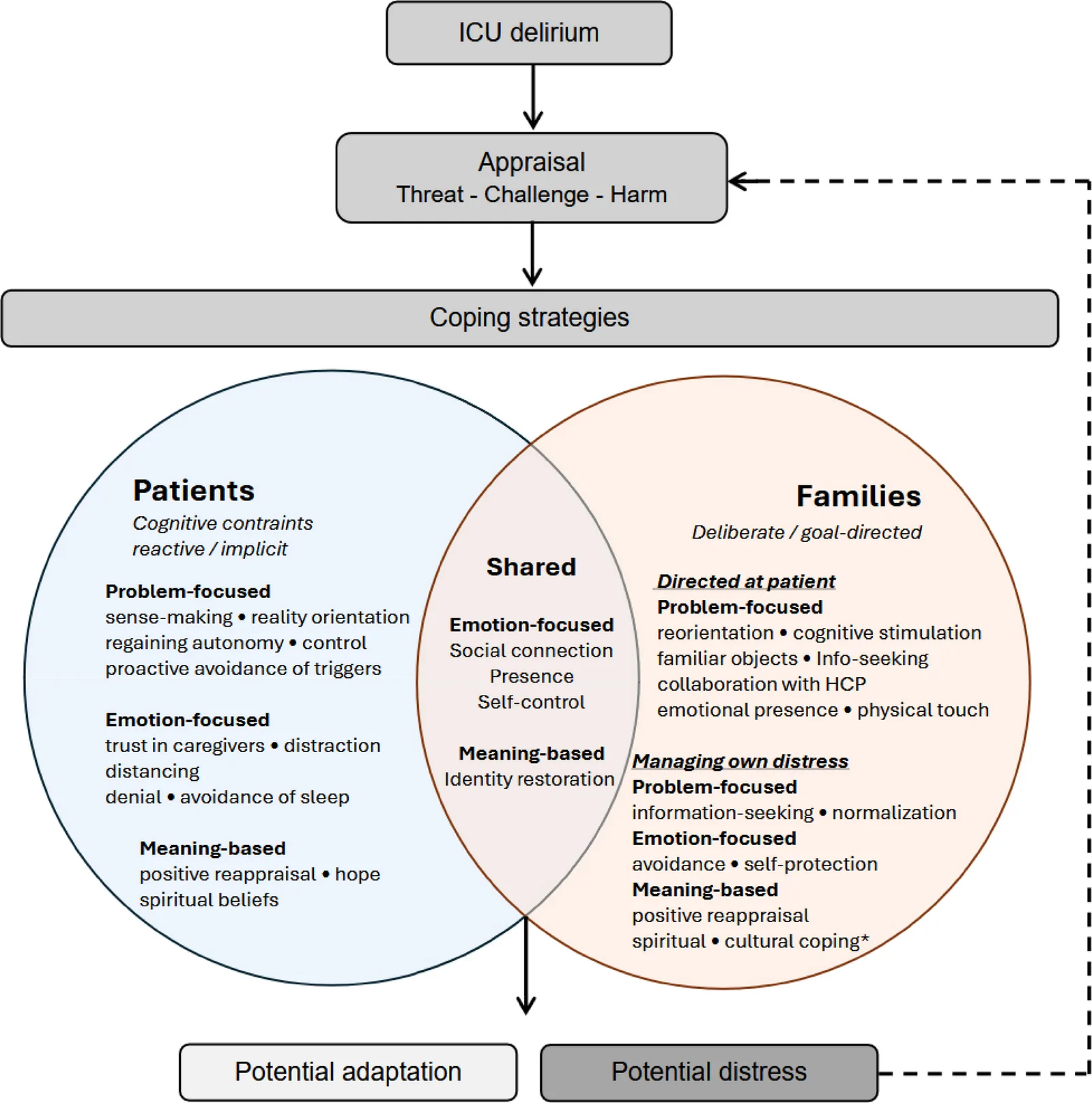
Conceptual synthesis of coping strategies used by ICU patients and family caregivers in response to ICU delirium. Coping strategies of ICU patients and their family caregivers in response to delirium, mapped onto the revised transactional model of stress and coping [19, 21]. Arrows indicate the direction of the coping process; the dashed arrow reflects reappraisal triggered by ongoing distress. Patient coping strategies encompass problem-focused, emotion-focused, and meaning-based responses, which may emerge reactively due to cognitive impairment imposed by delirium. Family coping strategies are directed both at supporting the patient and at managing their own distress, and are applied deliberately and goal-directedly. Shared strategies are represented in the overlapping zone. *Cultural coping strategies fall beyond the model's scope. Adapted from Folkman (1997) [21]. HCP - healthcare professionals; ICU - intensive care unit

The synthesis conducted in this review, oriented along the transactional model of stress and coping, enables a systematic understanding of the coping strategies employed by patients and families in response to ICU delirium, also revealing the model’s boundaries. This theoretical grounding represents an important step towards developing ICU delirium interventions better tailored to the psychosocial needs of both groups. Figure 2 illustrates the organization of the identified coping strategies within the revised model.

### Clinical implications and patient- and family-centred care (PFCC)

The findings of this review identify families as a primary active coping resource, consistent with the growing awareness of family participation in delirium management reflected in the ABCDEF (**A**ssess, prevent, and manage pain; **B**oth spontaneous awakening and breathing trials; **C**hoice of Analgesia and Sedation; **D**elirium assess, prevent, and manage; **E**arly Mobility and Exercise; **F**amily engagement/empowerment) bundle of the Society of Critical Care Medicine [40]. The most recently added component ‘F’ acknowledges this role formally. Among non-pharmacological interventions, family involvement and cognitive exercise have been shown to decrease the risk of delirium onset [41]. Delirium interventions implemented by family caregivers through their presence seem to have positive effects on patient outcomes, providing orientation, identity, and social connection [42].

Importantly, family participation in delirium care does not only benefit patients. Family caregivers of ICU patients with delirium are themselves predisposed to significant psychological burden, including anxiety, depression, and PTSD. Their active involvement in the care process has been shown to be one of the most effective preventions of these adverse psychological effects [17]. Family involvement in patient care therefore yields a bilateral benefit, consistent with the principles of PFCC, emphasized in the Family-Centred Care Guidelines of the Society of Critical Care Medicine [43]. Recently, digital communication tools have been shown to further facilitate family involvement in intensive care units, complementing in-person presence as a coping resource for both patients and families [44].

The coping strategies identified in this review provide a basis for developing targeted psychosocial interventions in ICU delirium care. For patients, fostering sense-making through regular, accessible explanations by healthcare professionals and the use of ICU diaries may support cognitive reorientation during and after delirium episodes [45]. Enabling small autonomous decisions, such as music selection or environmental preferences, may further support patients’ sense of control. For families, structured involvement in reorientation activities, guided by simple checklists or brief educational sessions, could translate the identified coping strategies into routine clinical practice. Given that family participation also reduces families’ own psychological burden, integrating psychological support for family caregivers into ICU care protocols is equally indicated. Finally, as coping processes extend beyond the ICU stay, continuity of psychosocial support into post-ICU recovery, addressing Post-Intensive Care Syndrome (PICS) and Post-Intensive Care Syndrome - Family (PICS-F), should be considered in future intervention development.

### Limitations

This review synthesized evidence on coping strategies of ICU patients and families for the first time. Nonetheless, certain limitations should be acknowledged. First, this review was designed as a mixed-methods systematic review; however, no quantitative studies meeting the eligibility criteria were identified, restricting the statistical generalization of the findings. This reflects the current absence of quantitative evidence on coping with ICU delirium, a gap that future research must address to refine evidence-based psychosocial support in clinical practice. The absence of quantitative data may partly reflect publication bias, as gray literature was not systematically searched. The mixed-methods framework was nonetheless retained, as the review was prospectively registered as an MMSR in prior to the search, and the absence of quantitative evidence is therefore a finding of the review rather than a post-hoc modification of the design. Correspondingly, the MMAT was selected as the quality appraisal tool given its consistency with the pre-registered protocol; it is acknowledged, however, that a checklist designed exclusively for qualitative studies may have offered greater sensitivity. Second, the included studies span from 1999 to 2025, a period of substantial changes in ICU practice, which may limit the comparability of findings across studies. Future research should therefore aim for more contemporary evidence. Third, coping was not the primary outcome in the majority of included studies; only Zhang et al. (2025) explicitly identified coping strategies as a named theme, though without an a priori theoretical framework guiding the analysis [32]. This limits the depth and specificity of the identified strategies. Studies with coping as a primary outcome are needed to deepen the understanding of this research area. Fourth, the transactional model of stress and coping was applied as an analytical framework to studies that did not originally adopt this theoretical basis. The identified coping categories may therefore reflect the structure of the model rather than emerging independently from the data. Future studies should consider an inductive approach to ensure that the full range of coping strategies employed by patients and families is captured. Fifth, the geographical distribution of the included studies was insufficient to represent a cross-cultural perspective, with no representation from low- or middle-income settings. Culturally sensitive instruments and diverse samples are needed. Furthermore, this review focused exclusively on adult ICU populations; paediatric and neonatal settings were excluded, despite family involvement being arguably even more central in these contexts. Future research should examine coping with ICU delirium in paediatric and neonatal settings. Finally, the evidence scope of this review is confined to the period of ICU hospitalization, whereas coping may unfold more broadly after discharge. Longitudinal studies encompassing PICS and PICS-F are therefore needed.

Future research should employ quantitative study designs using validated instruments such as the Coping Inventory for Stressful Situations (CISS) or the Brief Coping Orientation to Problems Experienced (Brief-COPE) [46, 47], alongside longitudinal approaches capturing PICS and PICS-F outcomes for post-ICU coping. The identified coping strategies should further inform the development of targeted psychosocial interventions for patients and families in delirium care.

## Conclusions

Applying the theoretical framework of Lazarus and Folkman, both patients - despite the cognitive constraints of delirium - and families emerged as active coping agents in response to ICU delirium, underlining the need to address both perspectives in clinical practice. The identified coping strategies provide a foundation for developing patient- and family-centred, coping-informed interventions, including structured family involvement in reorientation activities and continuity of psychosocial support into post-ICU recovery. In the future, quantitative research encompassing post-ICU coping and cultural diversity is needed. More broadly, recognizing patients and families as active coping agents offers a conceptual reorientation with direct implications for how psychosocial support in ICU delirium is designed and delivered.

## Supplementary Information

Below is the link to the electronic supplementary material.

Supplementary Material 1: the complete search strategy.

## Data Availability

All data generated or analyzed during this study are included in this published article and its supplementary information files.

## Declarations

The authors acknowledge the use of ChatGPT-5.5 (OpenAI) to support language editing during the revision process. The authors take full responsibility for the content of this manuscript.
