# Supplementary material for: Psychosocial coping with intensive care delirium: a mixed-methods systematic review of patient and family perspectives
Source: Crit Care. 2026 Aug 21;30:440. doi: 10.1186/s13054-026-06232-1 (PMC13495211; doi:10.1186/s13054-026-06232-1)
Supplement: Supplementary file 1 [file 13054_2026_6232_MOESM1_ESM.docx]

**Supplementary Material**

**Additional File 1: Search Strategy**

("intensive care units"[MeSH Terms] OR "intensive care units"[Title/Abstract] OR "icu"[Title/Abstract] OR "critical care"[MeSH Terms] OR "critical care"[Title/Abstract] OR "intensive care"[Title/Abstract] OR "critical illness"[MeSH Terms] OR "critical illness"[Title/Abstract] OR "ITU"[Title/Abstract] OR "CCU"[Title/Abstract]) 

**AND** ("Delirium"[MeSH Terms] OR "Delirium"[Title/Abstract] OR "acute brain dysfunction"[Title/Abstract] OR "encephalopathy"[Title/Abstract] OR "Cognitive Dysfunction"[MeSH Terms] OR "Cognitive Dysfunction"[Title/Abstract] OR "Sepsis-Associated Encephalopathy"[MeSH Terms] OR "septic encephalopathy"[Title/Abstract] OR "acute confusion"[Title/Abstract] OR "icu psychosis"[Title/Abstract]) 

**AND** ("positive adaptation"[Title/Abstract] OR "coping resources"[Title/Abstract] OR "meaning-making"[Title/Abstract] OR "psychological growth"[Title/Abstract] OR "narrative recovery"[Title/Abstract] OR "success story"[Title/Abstract] OR "recovery journey"[Title/Abstract] OR "success stor*"[Title/Abstract] OR "Lived experience"[Title/Abstract] OR "First-person perspective"[Title/Abstract] OR "Narrative accounts"[Title/Abstract] OR "coping skills"[MeSH Terms] OR "coping"[Title/Abstract] OR "rehabilitation"[Title/Abstract] OR "rehabilitation"[MeSH Terms] OR "social integration"[MeSH Terms] OR "Community Integration"[MeSH Terms] OR "social integration"[Title/Abstract] OR "Community Integration"[Title/Abstract] OR "Mental Health Recovery"[MeSH Terms] OR "Mental Health Recovery"[Title/Abstract] OR "Psychiatric Rehabilitation"[MeSH Terms] OR "Psychiatric Rehabilitation"[Title/Abstract] OR "Recovery of Function"[MeSH Terms] OR "Recovery of Function"[Title/Abstract] OR "Psychosocial Intervention"[MeSH Terms] OR "Psychosocial Intervention"[Title/Abstract] OR "Psychosocial Functioning"[MeSH Terms] OR "Psychosocial Functioning"[Title/Abstract] OR "Psychosocial Support Systems"[MeSH Terms] OR "Psychosocial Support Systems"[Title/Abstract] OR "Health Promotion"[MeSH Terms] OR "Health Promotion"[Title/Abstract] OR "Psychological First Aid"[MeSH Terms] OR "Psychological First Aid"[Title/Abstract] OR "Survivorship"[MeSH Terms] OR "Survivorship"[Title/Abstract] OR "Problem Solving"[MeSH Terms] OR "Problem Solving"[Title/Abstract] OR "adaptation, psychological"[MeSH Terms] OR "adaptation psychological"[Title/Abstract] OR "Episode of Care"[MeSH Terms] OR "Episode of Care"[Title/Abstract] OR "Population Health Management"[MeSH Terms] OR "Population Health Management"[Title/Abstract] OR "resilience, psychological"[MeSH Terms] OR "resilience psychological"[Title/Abstract] OR "Mental Health"[MeSH Terms] OR "Mental Health"[Title/Abstract] OR "Disease-Free Survival"[MeSH Terms] OR "Disease-Free Survival"[Title/Abstract] OR "psychology, positive"[MeSH Terms] OR "psychology positive"[Title/Abstract] OR "Hope"[MeSH Terms] OR "Hope"[Title/Abstract] OR "Sense of Coherence"[MeSH Terms] OR "Sense of Coherence"[Title/Abstract] OR "posttraumatic growth, psychological"[MeSH Terms] OR "posttraumatic growth psychological"[Title/Abstract] OR "Self Efficacy"[MeSH Terms] OR "Self Efficacy"[Title/Abstract] OR "Empowerment"[MeSH Terms] OR "Empowerment"[Title/Abstract] OR "Personal Satisfaction"[MeSH Terms] OR "Personal Satisfaction"[Title/Abstract] OR "Patient Satisfaction"[MeSH Terms] OR "Patient Satisfaction"[Title/Abstract] OR "Narration"[MeSH Terms] OR "Narration"[Title/Abstract] OR "health knowledge, attitudes, practice"[MeSH Terms] OR "health knowledge attitudes practice"[Title/Abstract] OR "Social Support"[MeSH Terms] OR "Social Support"[Title/Abstract] OR "Spirituality"[MeSH Terms] OR "Spirituality"[Title/Abstract] OR "Therapeutic Alliance"[MeSH Terms] OR "Therapeutic Alliance"[Title/Abstract] OR "experience"[Title/Abstract] OR "perception"[Title/Abstract] OR "perspective"[Title/Abstract] OR "attitude"[Title/Abstract] OR "interview"[Title/Abstract] OR "explor*"[Title/Abstract])

**AND** ("professional family relations"[MeSH Terms] OR "family"[MeSH Terms] OR "family"[Title/Abstract] OR "families"[Title/Abstract] OR "relatives"[Title/Abstract] OR "Patients"[MeSH Terms] OR "Patients"[Title/Abstract] OR "Caregivers"[MeSH Terms] OR "Caregivers"[Title/Abstract] OR "family-based"[Title/Abstract])

**NOT** "review"[Publication Type]
